# Data to model the effects of perceived telecommunication service quality and value on the degree of user satisfaction and e-WOM among telecommunications users in North Cyprus

**DOI:** 10.1016/j.dib.2019.104981

**Published:** 2019-12-16

**Authors:** Muneer Alrwashdeh, Ashraf Jahmani, Blend Ibrahim, Hasan Yousef Aljuhmani

**Affiliations:** a Department of Electronic Marketing and Social Media, Middle East University, Amman, Jordan; b College of Business Administration, Al Falah University, Dubai, United Arab Emirates; c Girne American University, School of Tourism and Hospitality Management, Department of Tourism, via Mersin 10, Kyrenia, North Cyprus, Turkey; d Faculty of Business and Economics/Centre for Management Research, Girne American University, via Mersin 10, Kyrenia, Cyprus (Northern), Turkey

**Keywords:** Service quality, Perceived value, User satisfaction, e-WOM, Mobile value-added services

## Abstract

This dataset is used to investigate a comprehensive model of user satisfaction and electronic word of mouth (e-WOM). Building on the perceived telecommunication service quality and perceived value dimensions in enhancing and improving users' satisfaction and e-WOM, we empirically investigated how the dimensions of service quality as a second-order construct and perceived value dimensions affect users’ satisfaction and e-WOM in the context of mobile operators in North Cyprus. The data were collected through a self-administered questionnaire at Girne American University. The dataset was empirically evaluated using a survey of 500 respondents regarding their perceptions of the service provided by the mobile telecom operator. Data analysis involved structural equation modelling (SEM) using Statistical Package for the Social Sciences (SPSS) 23 and Analysis of Moment Structures (AMOS) 24. First, we conducted exploratory factor analysis (EFA), confirmatory factor analysis (CFA), and structural modelling, followed by hypothesis testing. The outcomes obtained from this dataset indicated that perceived telecommunication service quality was positively related to perceived value dimensions (performance value and emotional value) and user satisfaction. At the same time, performance value, value for money, and social value were found to have a direct impact on user satisfaction. Specifically, there was a significant relationship between user satisfaction and e-WOM. The results may provide further insights into mobile value-added services.

**Table:** Specifications table

Subject	Business Management, Social media marketing, Marketing communication
Specific subject area	Service quality, Customer experience level, Customer satisfaction, Electronic word of mouth
Type of data	Tables, Figures, Images
How data were acquired	Data were mainly collected by distributing questionnaires to respondents who reported their patronage habits regarding Turkish mobile operators in North Cyprus.
Data format	Raw, analysed, descriptive, and statistical data
Parameters for data collection	To collect data, a self-administered questionnaire survey was conducted between April and May 2019 at Girne American University. The population comprised undergraduate and graduate students and the respondents were at least 17 years old. The questionnaire was composed of two sections. The first section was intended to capture each respondent's basic personal data. The second section measured the relationship between service quality, perceived value, e-WOM, and customer satisfaction regarding mobile phone companies Telsim and Turkcell, which are Turkish companies operating in North Cyprus.
Description of data collection	Due to the superiority of performance-based measures, factors such as service quality were operationalised using the SERVPERF model. The construct of service quality was initially grounded on the three examining dimensions (content quality, management and customer service, and system reliability and connection quality). Perceived value is a trade-off between what customers receive, such as quality, benefits, and utilities, and what they sacrifice, such as price, opportunity cost, time, and efforts, which are instrumental in enhancing and improving customer satisfaction and e-WOM.
Data source location	Girne American University in the Turkish Republic of Northern Cyprus (TRNC)
Data accessibility	https://doi.org/10.17632/m92kjz7tm8.2

**Table:** 

**Value of the Data** • The dataset is useful because it measures the effect of perceived telecom service quality from three different perspectives (management and customer service, content quality, system reliability and connection quality) and the dimensions of perceived value (performance value, value for money, emotional value and social value) on user satisfaction and e-WOM. • This dataset may prove to be valuable background information for mobile value-added services to improve their service quality in order to enhance users' perceived value and to increase their customer experience level within the company. • The dataset could be used for further analysis to test the mediation effect of perceived value dimensions (performance value, value for money, emotional value, and social value) on the relationship between perceived telecommunication service quality and user satisfaction. At the same time, future research could investigate the indirect relationship between perceived value dimensions and e-WOM through user satisfaction. • This dataset bridges a gap in the literature by investigating whether user satisfaction affects the e-WOM for mobile telecom services. Therefore, this dataset also corroborates that service quality and the dimensions of perceived value are an important predictor of customer satisfaction. By extending our research model, future research focusing on the outcomes of customer satisfaction and e-WOM could provide a holistic picture of the nature of the relationship between these two variables, which will produce interesting results.

## 1 Data

**Fig. 1:**
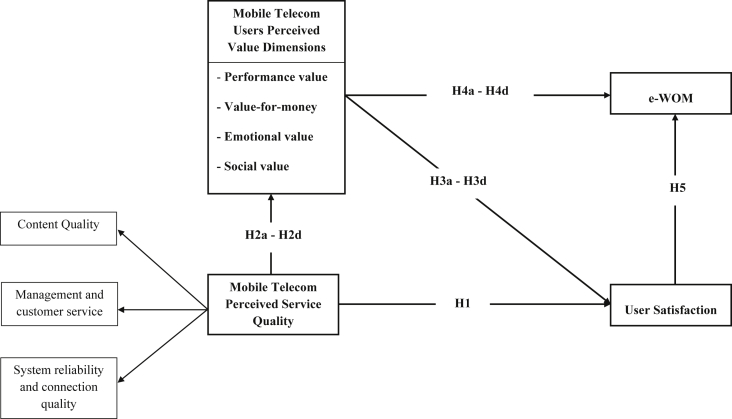
Proposed research model.

**Fig. 2:**
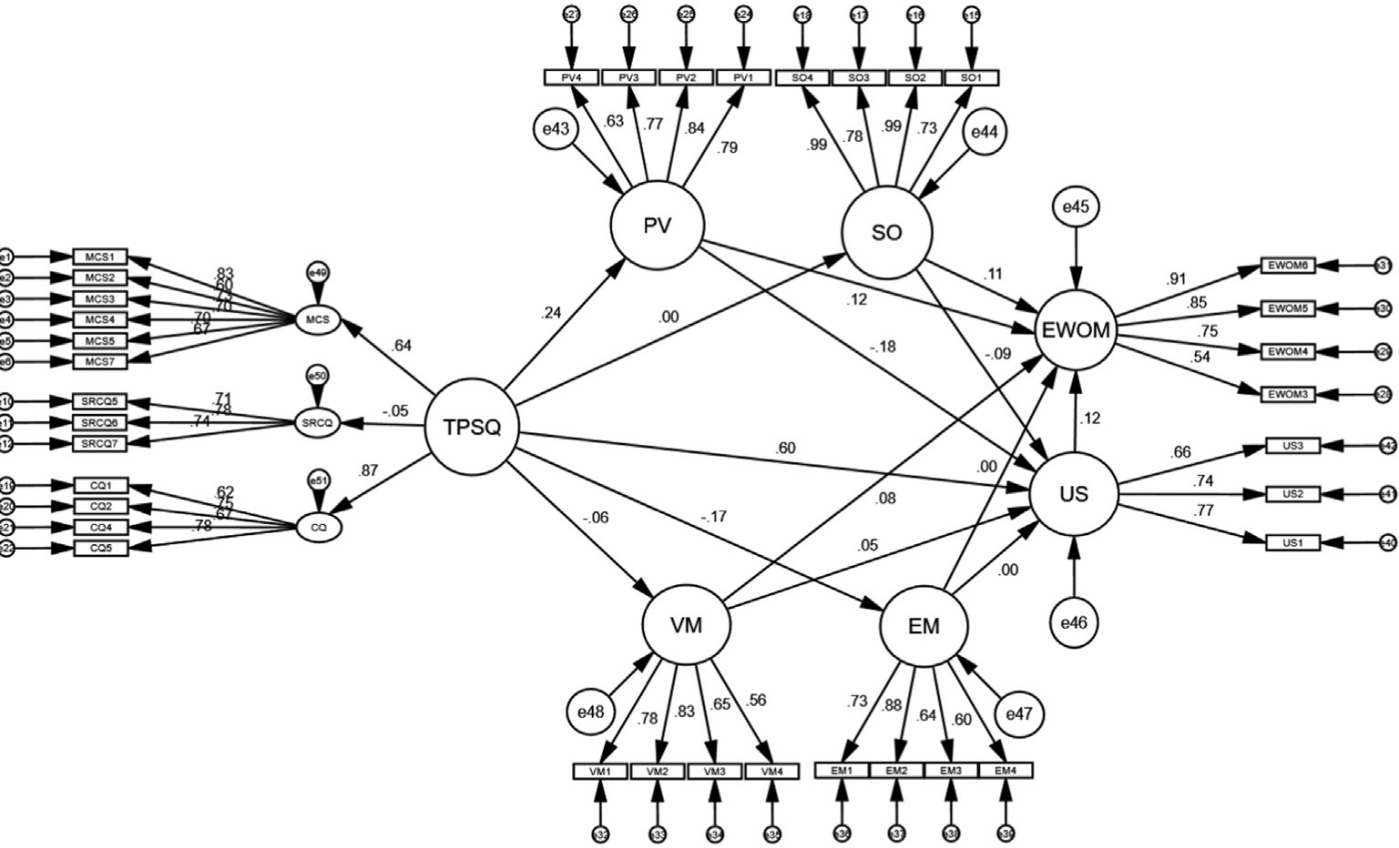
Structural model. Note: Telecom perceived service quality (TPSQ), management and customer services (MCS), performance value (PV), content quality (CQ), emotional value (EM), value for money (VM), system reliability and connection quality (SRCQ), user satisfaction (US), social value (SO), and electronic word of mouth (EWOM).

The data collected were compiled according to literature and users' and experts' opinions. After the draft questionnaire was completed, a pre-test was performed on experts and users familiar with mobile service providers to modify items with ambiguous expressions. Therefore, respondents could understand the questions in the formal survey and the content validity of the questionnaire could be ensured. All items were assessed using five-point Likert scales from 1 ‘strongly disagree’ to 5 ‘strongly agree’. A total of 390 valid questionnaires were collected; 25 were excluded due to incomplete responses, and 365 valid and useable questionnaires were obtained for data analysis. IBM, SPSS, and AMOS software were used to interpret and summarise the data, and descriptive statistical techniques were utilised to calculate the means, frequency, percentage, and standard deviations of the responses. Given the nature of the proposed conceptual research model (Fig. 1), a structural equation modelling (SEM) technique was adopted. SEM is a common method used by practitioners in marketing research [1,2], especially to assess causal research models and research hypotheses. SEM allows researchers to jointly test interrelated hypotheses by evaluating the relationships between multiple independent and dependent constructs in a structural model [3]. AMOS software version 24 was used to estimate model measurements and structural models. The data are summarized through two figures and four tables. Table 1 presents the research constructs and items included in the questionnaire. Table 1 also shows the reliability and validity of the measurement models. In addition, Table 2 reported the results of model fit measures. Furthermore, Table 3 presents the means, standard deviations and correlation matrix among the data constructs. Fig. 2 and Table 4 represent the structural model and data results, respectively.

**Table 1:** Measurement accuracy assessment.

Experiential component	Items	EFA Factor loading	CFA standardised loading	Cronbach Alpha values	CR	AVE
**Mobile Telecom Perceived Service Quality**						
Content quality	CQ1. ‘This telecom company service provides complete content’.	0.825	0.623	0.84	0.80	0.502
	CQ2. ‘This telecom company service provides appropriate content’.	0.627	0.751			
	CQ3. ‘This telecom company service provides important content’. *	0.786	–			
	CQ4. ‘This telecom company service provides fashionable content’.	0.670	0.670			
	CQ5. ‘This telecom company service provides regularly updated content’.	0.810	0.781			
	CQ6. ‘I can fully understand the content provided by this telecom company’. *	–	–			
Management and customer service	MCS1. ‘This telecom company provides diversified services’.	0.841	0.833	0.86	0.86	0.505
	MCS2. ‘This telecom company provides multiple tariff options’.	0.688	0.602			
	MCS3. ‘This telecom company provides good post-services’.	0.738	0.734			
	MCS4. ‘I can easily alter my contract with this telecom company’.	0.788	0.700			
	MCS5. ‘When I alter contract, the telecom company still has a friendly attitude’.	0.744	0.701			
	MCS6. ‘When any problem occurs, the telecom company can instantly deal with it’. *	0.657	–			
	MCS7. ‘This telecom company provides a FAQ for service provides’.	0.690	0.675			
System reliability and connection quality	SRCQ1. ‘This telecom company's system is stable’. *	0.726	–	0.82	0.79	0.551
	SRCQ2. ‘Errors seldom occur in this telecom company system’. *	0.646	–			
	SRCQ3. ‘This telecom company provides effective links’. *	0.669	–			
	SRCQ4. ‘I can easily return to the previous screen’. *	0.656	–			
	SRCQ5. ‘It does not take too much time to download the information I need’.	0.702	0.709			
	SRCQ6. ‘It does not take too much time to load the links I click on’.	0.800	0.780			
	SRCQ7. ‘This telecom company system can instantly react to the data I input’.	0.764	0.738			
**Mobile Telecom Users Perceived Value Dimensions**						
Performance value	PV1. ‘This telecom company has consistent quality’.	0.860	0.788	0.84	0.85	0.579
	PV2. ‘This telecom company's services are well designed’.	0.866	0.841			
	PV3. ‘This telecom company has an acceptable standard of quality’.	0.798	0.766			
	PV4. ‘This telecom company offers consistent quality’.	0.768	0.633			
Value for money	VM1. ‘The services provided by this company are reasonably priced’.	0.813	0.784	0.80	0.80	0.504
	VM2. ‘This telecom company offers value for money’.	0.851	0.822			
	VM3. ‘This telecom company offers a good service for the price’.	0.770	0.645			
	VM4. ‘This telecom company is economical’.	0.703	0.558			
Emotional value	EV1. ‘Using this telecom company makes me feel relaxed’.	0.748	0.723	0.80	0.81	0.516
	EV2. ‘I enjoy using this telecom company’.	0.846	0.887			
	EV3. ‘Using this telecom company makes me feel good’.	0.763	0.634			
	EV4. ‘Services offered by this telecom company give me pleasure’.	0.796	0.592			
Social value	SV1. ‘The fact I use this telecom company makes a good impression on other people’.	0.644	0.726	0.88	0.93	0.776
	SV2. ‘Using this telecom company brings me social approval’.	0.973	0.993			
	SV3. ‘Using this telecom company helps me feel accepted’.	0.841	0.776			
	SV4. ‘Using this telecom company improves the way I am perceived’.	0.977	0.994			
**User Satisfaction**	US1. ‘I am satisfied with the services provided by this telecom company’.	0.811	0.767	0.75	0.77	0.522
	US2. ‘I think this telecom company has successfully provided services’.	0.776	0.737			
	US3. ‘The service provided by this telecom company is better than expected’.	0.791	0.659			
**Electronic Word of Mouth**	e-WOM1. ‘To make sure that I buy the right products or brands, I often read online reviews written by fellow members on social networks’. *	–	–	0.81	0.85	0.599
	e-WOM2. ‘To choose the right products or brands, I often consult online reviews of products and brands provided by other fellow members on social networks’. *	–	–			
	e-WOM3. ‘The information that I spread on social networks regarding products and brands usually influences the opinion of other members’.	0.714	0.536			
	e-WOM4. ‘I always publish my experiences with products and brands on social networks at the request of other members’.	0.830	0.753			
	e-WOM5. ‘I am willing to share my experiences with products and brands with other members on social networks’.	0.867	0.850			
	e-WOM6. ‘I try to share my experiences with products and brands with other fellow members on social networks more effectively’.	0.892	0.905			

Note: Average variance extracted (AVE), composite reliability (CR), exploratory factor analysis (EFA), confirmatory factor analysis (CFA), * Items deleted.

**Table 2:** Model fit measures.

Measures	Recommended criteria	Measurement model	Structural model	References
CMIN	–	910.416	49.845	[9,10]
DF	–	559.000	19.000	
X^2^/d.f. (p-value)	<3	1.63 (0.000)^a^	2.62 (0.000)^a^	
CFI	>0.9	0.94	0.93	
GFI	>0.8	0.88	0.97	
AGFI	>0.8	0.86	0.93	
SRMR	<0.08	0.05	0.05	
RMSEA	<0.08	0.04	0.07	
PClose	>0.05	0.998	0.102	

a For a larger sample size, significant p-values are expected [11].

**Table 3:** Means, standard deviations, and correlations matrix among the data constructs.

*n* = *365*	Mean	St. Deviation	1	2	3	4	5	6	7	8	9
1. MCS	2.50	0.834	***0.711***								
2. SRCQ	3.17	0.651	0.014	***0.743***							
3. SO	1.44	0.667	−0.024	0.043	***0.881***						
4. CQ	2.23	0.441	0.557***	−0.057	0.025	***0.709***					
5. PV	3.16	0.625	0.234***	0.080	−0.060	0.166*	***0.761***				
6. EWOM	1.53	0.521	0.055	−0.080	0.095†	0.106†	0.107†	***0.774***			
7. VM	2.12	0.757	−0.017	−0.036	−0.009	−0.078	−0.028	0.082	***0.710***		
8. EM	2.86	0.480	−0.122*	−0.081	0.103†	−0.149*	−0.071	−0.027	−0.219***	***0.718***	
9. US	2.36	0.525	0.323***	−0.109	−0.086	0.496***	−0.036	0.100	0.019	−0.111†	***0.723***

Note: Diagonals (in bold and italics) represent the average variance extracted (AVE), while the other matrix entries represent the shared variance (the squared correlations). Management and customer services (MCS), performance value (PV), content quality (CQ), emotional value (EM), value for money (VM), system reliability and connection quality (SRCQ), user satisfaction (US), social value (SO), and electronic word of mouth (EWOM). Correlation is significant at †p < 0.100, *p < 0.050, ***p < 0.001.

**Table 4:** Outcomes of structural equation model analysis.

Path	Hypothesis	Estimate	CR	*P*	Result
TPSQ → User satisfaction	H1	0.829	19.406	***	Supported
TPSQ → Performance value	H2a	0.359	5.523	***	Supported
TPSQ → Value for money	H2b	−0.132	−1.620	0.105	Not Supported
TPSQ → Social value	H2c	0.018	0.250	0.803	Not Supported
TPSQ → Emotional value	H2d	−0.211	−4.133	***	Supported
Performance value → User satisfaction	H3a	−0.206	−6.500	***	Supported
Emotional value → User satisfaction	H3b	0.048	1.200	0.230	Not Supported
Value for money → User satisfaction	H3c	0.062	2.449	0.014	Supported
Social value → User satisfaction	H3d	−0.100	−3.516	***	Supported
Performance value → e-WOM	H4a	0.113	2.689	0.007	Supported
Emotional value → e-WOM	H4b	0.005	0.083	0.934	Not Supported
Value for money → e-WOM	H4c	0.065	1.881	0.060	Not Supported
Social value → e-WOM	H4d	0.098	2.480	0.013	Supported
User satisfaction → e-WOM	H5	0.134	2.661	0.008	Supported

Note: TPSQ: Telecom perceived service quality; e-WOM: Electronic word of mouth; *** Statistically significant at *p* _ 0.001.

## 2 Experimental design, materials, and methods

We employed a quantitative cross-sectional approach. Following Podsakoff et al. [4], participants were asked to voluntarily participate and were assured of confidentiality; additionally, information about the research intent was provided and participants were also informed that there were no right or wrong answers and that they should answer as honestly as possible. According to Kaden [5], a solid sample size for marketing research is around 300. However, based on the sampling calculator suggestion, a sample size of 400 is adequate. In order to increase reliability, 500 questionnaires were distributed using a face-to-face (hand-delivered) method. Data were collected via the self-administered questionnaire between April and May 2019 at Girne American University, which has 17,751 students. The population of this dataset consisted of undergraduate and graduate students, the respondents were 17 years old and older. A simple random sampling technique was employed. Following Bush and Hair [6], data were collected at different times of the day and on different days of the week.

As a first step, exploratory factor analysis (EFA) was conducted using SPSS version 23, the principal component method was applied, and oblique Promax with Kaiser Normalisation was employed. The results of EFA are reported in Table 1 above. To retain the interrelated items, 0.4 was taken as the minimum factor loading. Three items related to CQ item 6 and e-WOM items 1 and 2 were deleted during EFA because the low factor loading was less than the acceptable threshold of 0.4. In addition, the extracted variance values ranged from 13.5 to 63.0% among the constructs. The cumulative variance explained value was above the acceptable cut-off point of 50%, which seemed to be satisfactory and were retained for our future analysis of the CFA and structural model [7]. Cronbach's alphas were determined to test the validity and reliability of our collected data; all factors were above the acceptable cut-off point of 0.70 [7].

As a next step, confirmatory factor analysis (CFA) was carried out. As noted by Harrington [8], CFA is a statistical technique used to verify the factor structure of a set of observed variables. Bagozzi and Yi [9] added that CFA assists scholars and researchers in identifying and determining construct validity (i.e. convergent, discriminant, and nomological validity). CFA was conducted on the overall model using AMOS version 24. We used different criteria and thresholds in conducting the model fit measures such as comparative fit index (CFI), goodness of fit index (GFI), adjusted goodness of fit (AGFI), square error of approximation (RMSEA), and standardised root mean square residual (SRMR). Table 2 summarises the acceptable thresholds and results of model fit measures.

Furthermore, to test the convergent validity, CFA standardised factor loadings (λ) were checked. Construct reliability (CR) and average variance extracted (AVE) were also checked based on the CFA model measurement. Table 1 summarises the standardised factor loadings, CR, and AVE. The CR values were above the acceptable threshold of 0.70 [12]. The standardised factor loading values were above the acceptable cut-off point of 0.50. Finally, AVE values exceeded the acceptable cut-off points of 0.50 [12]. These values confirmed the construct and convergent reliability of the data. Table 3 presents the correlation matrix among the data constructs.

### 2.1 Path model

To interpret and estimate the data results, structural equation modelling based on the AMOS version 24 statistics program was used to test the data hypotheses. Fig. 2 and Table 4 represent the structural model and data results, respectively. A first step in constructing a structural model is determining a good model fit measure. Table 2 reported the results of model fit measures. Our research model met all the criteria for a good model fit [9,13]. All these procedures have been used by Alrwashdeh et al. [14] and Ibrahim and Aljarah [15] in recent research conducted in the same field and context of North Cyprus.

First, a scale for measuring perceived telecommunication service quality was proposed. Through exploratory and confirmatory factor analyses, we identified three dimensions of perceived telecom service quality, including content quality, management and customer service, and system reliability and connection quality. That is, perceived service quality was used to measure the service quality of mobile service providers and was measured using 20 items adapted from Ref. [16], which dealt with the value-added of mobile services in Taiwan. These items were also modified to fit the context of telecommunication providers. Kuo et al. [16] found that perceived service quality dimensions were positively associated with perceived value and customer satisfaction. In addition, perceived value dimensions (performance value, value for money, emotional value, and social value) were measured using 16 items adapted from Ref. [17]. These items were also modified to wording appropriate for the context of mobile service providers. In their study on a mobile application in Taiwan, Hsu and Lin [18] successfully used a recent version of this scale and found that, rather than performance value, value for money, and social value, perceived value in general and emotional value in particular have a significant effect on user satisfaction. In the current dataset, user satisfaction is defined ‘*as the total consumption perception of consumers’* when using mobile service providers [[16], p. 889]. User satisfaction was measured using three items adopted from Lin and Wang [19]. Finally, e-WOM was measured using six items adapted from Bambauer-Sachse and Mangold [20]. A recent version of this scale was successfully used by Jalilvand and Samiei [21]. The final instrument depicted in Table 1 showed adequate reliability and validity of the measurement model. Further, we empirically investigated how the dimensions of telecom service quality as a second-order construct and perceived value dimensions affect users' satisfaction in the context of mobile operators in North Cyprus. Perceived telecommunication service quality and value dimensions (performance value, value for money, and social value) are associated with user satisfaction. At the same time, perceived telecom value dimensions (performance value and social value) directly influence e-WOM and there is a positive relationship between user satisfaction and e-WOM.
