# Supplementary material for: Data to model the effects of perceived telecommunication service quality and value on the degree of user satisfaction and e-WOM among telecommunications users in North Cyprus
Source: Data Brief. 2019 Dec 16;28:104981. doi: 10.1016/j.dib.2019.104981 (PMC6931100; doi:10.1016/j.dib.2019.104981)
Supplement: Multimedia component 1 [file mmc1.pdf]

Language Editing Services

Registered Office:
Elsevier Ltd
The Boulevard, Langford Lane,
Kidlington, OX5 1GB, UK.
Registration No. 331566771

To whom it may concern

The paper "Data to Model the Effect of Telecommunication Perceived Service Quality and Perceived Value on the Degree of User Satisfaction and e-WOM: Context of North Cyprus Telecommunications Users" by Hasan Yousef Aljuhmani was edited by Elsevier Language Editing Services.

Kind regards,

Elsevier Webshop Support

(This is a computer generated advice and does not require any signature)
